# A global bibliometric and visual analysis of research on premature ovarian failure: Based on the perspective of stem cells

**DOI:** 10.1097/MD.0000000000038399

**Published:** 2024-05-31

**Authors:** Zhiguo Xu, Yi Zhu, Lefeng Liu, Chao Liu, Zhilong Dong

**Affiliations:** a Department of Pharmaceutical Engineering, School of Life and Health Sciences, Huzhou College, Huzhou, Zhejiang Province, China; b School of Life Sciences and Technology, Tongji University, Shanghai, China; c Xiehe Union East China Stem Cell & Gene Engineering Co., Ltd, Huzhou, Zhejiang Province, China; d Shaoxing University Yuanpei College, Shaoxing, Zhejiang Province, China; e Huzhou University, Huzhou, Zhejiang Province, China; f Tianjin Cord Blood Bank, National Industrial Base for Stem Cell Engineering Products, Tianjin, China; g State Key Laboratory of Medicinal Chemical Biology, Tianjin Key Laboratory of Protein Sciences and College of Life Sciences, Nankai University, Tianjin, China.

**Keywords:** Citespace, immunomodulation, premature ovarian failure, stem cell, visual analysis

## Abstract

Premature ovarian failure (POF), a condition influenced by genetic and immune factors, remains incurable despite years of intensive research and significant efforts. This persisting challenge underscores the urgency to address this escalating health concern. Fortunately, stem cell regenerative medicine has emerged as a promising avenue for developing therapeutic strategies and innovative treatments for POF. Bibliometric analysis, renowned for its objectivity, systematic approach, and comprehensive coverage of a given field, has yet to be applied to the study of stem cell research in POF. This study used CiteSpace software to assess contributions and co-occurrence relationships among various countries/regions, institutes, journals, and authors. This approach also allowed us to identify research hotspots and promising future trends within this field. Additionally, we generated visualizing maps utilizing the Web of Science Core Collection (WOSCC) and PubMed publications. By providing valuable information and references, we aim to enhance the understanding of the challenges involved in translating stem cell regeneration into clinical therapeutic potential for POF. Furthermore, our analysis and findings guide researchers and clinicians, facilitating future collaborative research and clinical intervention efforts.

## 1. Introduction

Premature ovarian failure (POF) is a syndrome caused by genetic factors, immune factors, and other causes of reduced follicular pool reserve or follicular dysfunction in the ovary, the etiology of which is unclear.^[1]^ Age <40, amenorrhea 6 months, and blood follicle-stimulating hormone (FSH) levels > 40 mIU/mL are the generally accepted diagnostic criteria for POF. Frequently accompanied by several perimenopausal symptoms, including dry skin, mucous membranes, hot flashes, night sweats, hair loss, sleep difficulties, irritability, decreased libido, lowered estradiol levels on clinical examination, elevated gonadotropin levels, scanty menstruation or amenorrhea.^[2–4]^ The number of infertile couples has steadily increased over the past 20 years, with age-related infertility rising at a significant rate. POF poses a potential threat, with the prevalence of clinical POF reaching 1% in people aged 35 to 40 and approximately 1 case per 1000 in people aged 18 to 25.^[5]^ The risk of POF before the age of 40 years is around 1% and its prevalence varies with age. Prevalence is 1:10,000 at the age of 18 to 25 years, 1:1000 at age 25 to 30 years, and 1:100 at 35 to 40 years. Given the worldwide delay in the age of childbirth, the decline in fertility rates, and the likelihood that POF plays a significant role in reduced fertility or even infertility, it has emerged as a crucial factor contributing to the global fertility decline, particularly among highly educated individuals.^[6,7]^ However, women with POF experience complex clinical symptoms and unfavorable outcomes (such as amenorrhea, hypomenorrhea, vasodilatory instability, hot flashes, night sweats, sleep disturbances, vulvovaginal atrophy, altered urinary frequency, difficulty with sexual intercourse, and low libido) that significantly affect their quality of life. These conditions include cognitive dysfunction, psychological disorders, osteoporosis, autoimmune diseases, and cardiovascular diseases. POF significantly impacts a patient quality of life, which is why public health receives so much attention from the general population. Since POF cannot be cured, pharmacologists and scientific researchers have struggled to find effective treatments for POF.^[5]^ Psychotherapy, androgen or dehydroepiandrosterone supplementation, herbal therapy, dietary and exercise modification, immunomodulation, and psychotherapy are common therapies for POF; nevertheless, none can fundamentally alter ovarian function and satisfy fertility goals.^[5,8]^ Hormone replacement treatment (HRT)^[9,10]^ is clinically successful in lowering osteoporosis and cardiovascular disease risk, treating menopausal symptoms, and enhancing patients’ quality of life. HRT, however, may increase the chances of developing breast or endometrial cancer.^[11]^ A new way of treating POF is the ultra-cryopreservation of ovarian tissue. However, cryopreserved ovarian tissue still has a lot of issues, including challenges with natural conception and a low survival rate.^[12]^ In recent years, researchers have been looking into new treatment options to prevent POF patients from experiencing negative side effects. These include using mitochondrial replacement therapy, artificial ovaries, artificial gametes, frozen ovarian tissue transplantation, platelet-rich plasma infusion, and artificial ovaries.^[13,14]^ Stem cell therapy has excellent efficacy in increasing FSH and luteinizing hormone levels, activating primary follicles and encouraging their transformation, as well as promoting tissue recovery and neovascularization. Therefore, POF patients now have a new hope for treatment.

Despite the wealth of published works on stem cell therapy for POF, there has been no systematic visualization and evaluation of published results in this area in the literature until now. Compared with traditional reviews and systematic reviews, bibliometrics is the use of mathematical and statistical methods to quantitatively analyze a large amount of literature in a research field to reveal many aspects and research trends in the field, which can more intuitively show the research content and hotspots in the field. The data analysis results can predict future research trends, thus focusing and leading future research directions.^[15,16]^ CiteSpace is an effective method and tool for analyzing large-scale data by forming knowledge graphs for quantitative information data analysis and visualization, which can visualize research hotspots and evolutionary processes in various fields and predict development trends. This paper is the first comprehensive scientometric study on stem cells and POF using CiteSpace. It aims to summarize the scientific progress and current hotspots in stem cells and POF and provide specific references for future research.^[17]^

## 2. Materials and methods

### 2.1. Data sources and search strategy

Data for this study were retrieved from Web of Science Core Collection (WOSCC) and PubMed on May 18, 2023. The following approaches were used to employ the MeSH and entrance words separately or jointly: All fields are equal to (“premature ovarian failure” AND “Stromal Cell” OR “Mesenchymal Stromal Cell” OR “Mesenchymal Progenitor Cell” OR “Stromal Cell, Mesenchymal” OR “induced pluripotent stem” OR “Amniotic epithelial cells” OR “Multipotent Mesenchymal Stromal Cell” OR “Stem cell” OR “Stem cells”). In the WOSCC database, we refined the documents according to the following criteria: (Article AND Review) AND language: (English). Meanwhile, we searched on PubMed according to the above search, and 114 publications were obtained. Two researchers (Z.-G.X. and Y.-Z.) retrieved and evaluated the publications. Until a consensus was obtained, disagreements were discussed with a third investigator (L.-F.L.).

### 2.2. Inclusion criteria

Peer-reviewed, previously published literature original works on POF and stem cell research, including basic and clinical studies; reviews of POF and stem cell research; articles found in the WOSCC and PubMed.

### 2.3. Exclusion criteria

Articles that were hand-collected or collected over the phone; papers that were unofficially published; conference abstracts and proceedings; duplicate publications; and unconnected articles.

### 2.4. Data analysis

**Figure 1.**
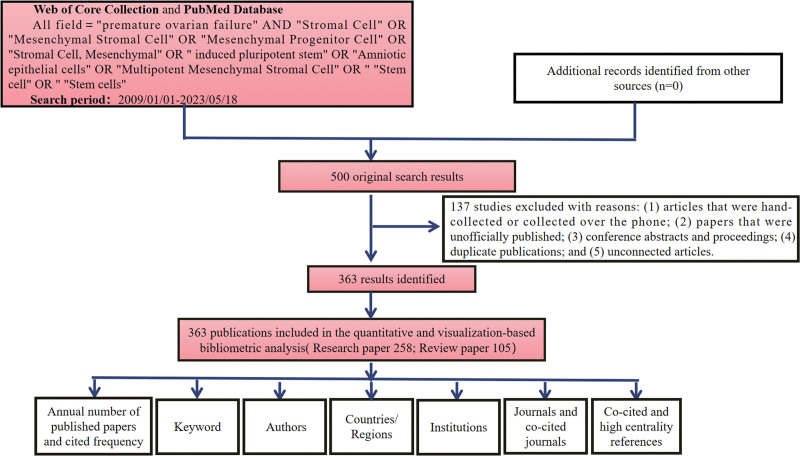
The retrieval strategy used in this study and methods of bibliometric analysis.

All valid records obtained from WOSCC and PubMed were translated to CiteSpace v.6.1.R6, 64-bit (Drexel University, Philadelphia) and Microsoft Excel 2003 for visual evaluation.^[18]^ Microsoft Excel 2003 (version 16.46) was used to conduct a simple descriptive analysis (such as the number of publications each year, papers with the most citations, etc). The distribution of nations/regions, authors, journals, keyword cluster analysis, co-cited references, and high-centrality references were visually analyzed using the CiteSpace software. Figure 1 shows specific enrollment and analysis techniques in detail.

## 3. Results

### 3.1. Analysis of the publication characteristics and citation trends

**Figure 2.**
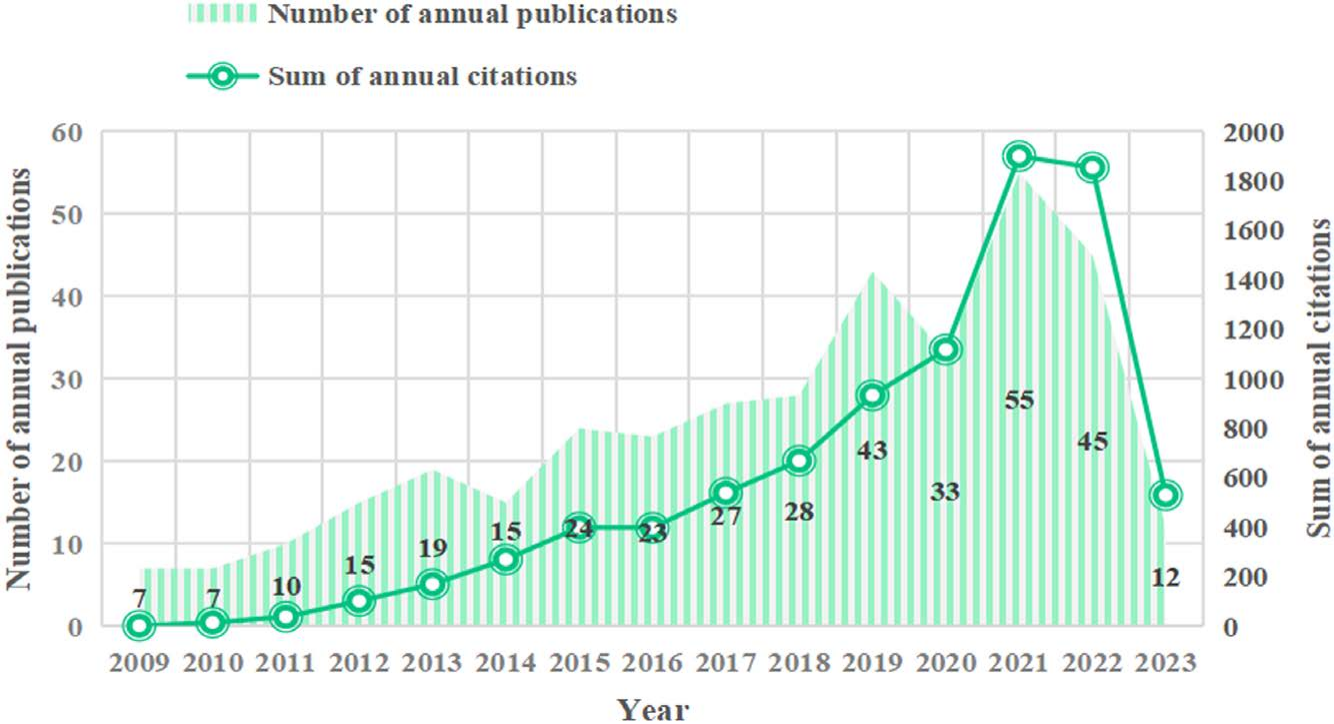
Distribution of publications and citations from different yr.

The temporal variations in a study area publication count can indicate how quickly that topic develops. The quantity of citations is a crucial evaluation metric for academic influence and quality. This study consisted of 363 papers in total. They included 105 (31.3%) review papers and 258 (68.7%) research papers. Figure 2 displays the total number of publications published over 15 years. From 2009 to 2023, the overall trend of articles on stem cells and POF is upward. This research can be separated into 3 phases depending on the number of annual publications and citations. Initial exploration (2009–2011), start-up (2012–2017), and rapid development (2018–2023) are the first, second, and third phases, respectively. By injecting young adult bone marrow-derived stem cells into female mice between 2009 and 2011, Kaisa Selesniemi et al at Harvard Medical School laid the foundation for stem cell therapy for POF by delaying age-related POF and improving offspring survival. However, the field received little attention from academics at this time. The number of publications showed a varying upward trend between 2012 and 2017, and the number of citations significantly grew, indicating that the work is gradually attracting the attention of academics. Between 2018 and 2022, the study advanced quickly, peaking in 2021 for both publications and citations.

### 3.2. Analysis of keyword co-occurrence and clustering analysis related to research hotspots

**Figure 3.**
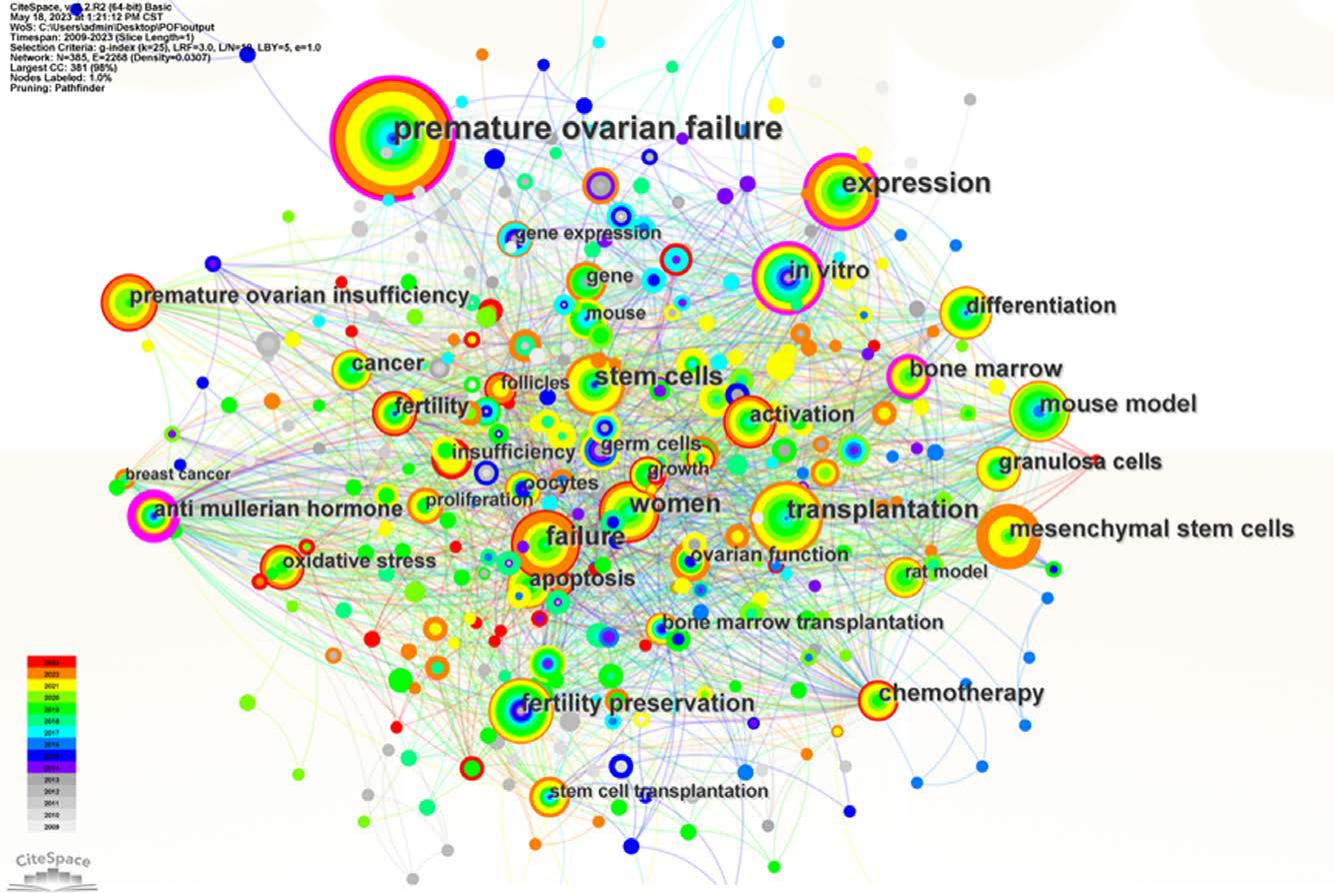
Keywords co-occurrence analysis of global research on stem cells in POF from 2009 to 2023. POF = premature ovarian failure.

Keywords are a high summary and overview of the content of the literature, mainly reflecting the research theme of the literature. They can be used to understand the central idea of literature to be presented in an overall way. The keywords with high centrality and frequency indicate the research hotspots that are of general interest to researchers over some time. In addition to the keywords “POF” and “stem cell,” which are the most basic terms in the field of ovarian failure and stem cell research, the top 15 high-frequency keywords are expression (n = 65), failure (n = 56), transplantation (n = 54), women (n = 48), mesenchymal stem cells (n = 40), chemotherapy (n = 40), bone marrow (n = 38), in vitro (n = 35), fertility preservation (n = 35), mouse model (n = 34), differentiation (n = 31), anti-mullerian hormone (n = 30), granulosa cells (n = 29), activation (n = 29), fertility (n = 29), apoptosis (n = 26), see Figure 3.

**Figure 4.**
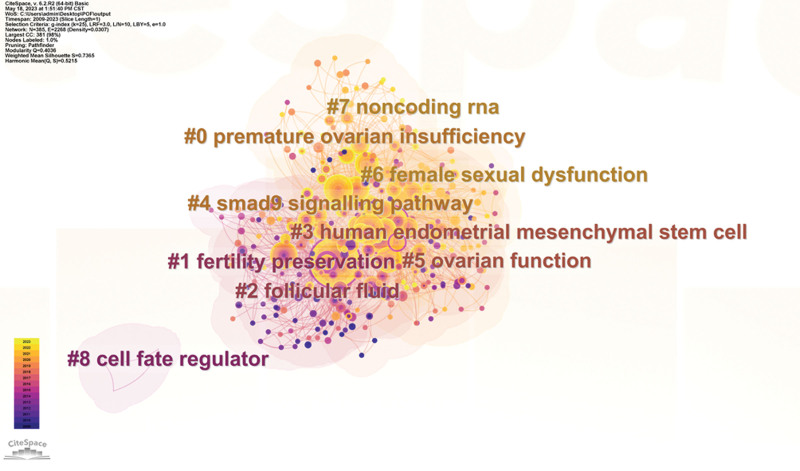
Clustering map of keyword co-occurrences in stem cells research in POF. POF = premature ovarian failure.

This study employed a log-likelihood ratio to cluster the high-frequency keywords to more thoroughly assess the knowledge structure of research hotspots connected to stem cells and POF and to investigate the combinatorial classification of keywords. The cluster labels serve as the primary focus of this field study, and the cluster ordinal number (#) is inversely correlated with cluster size, meaning that the smaller the cluster number, the more extensive the scope of research in the literature falling under this cluster and the greater the research hotspot. As shown in Figure 4, Modularity Q = 0.4036 is >0.3, indicating that this clustering is significant. Mean Sihouette = 0.7365, which is >0.7, suggesting that the results of this clustering are convincing. The keyword clustering graph showed several clusters overlapping, indicating that the clusters are closely related and that while stem cells vary in research relevant to POF, the topics are tightly concentrated and interconnected. A total of 9 clustering modules were obtained in this study (Fig. 4), and the 9 large-scale clustering modules were #0 premature ovarian insufficiency (POI), #1fertility preservation, #2 follicular fluid, #3 human endometrial mesenchymal stem cell, #4 smad9 signaling pathway, #5 ovarian function, #6 female sexual dysfunction, #7 noncoding RNA and #8 cell fate regulator.

**Figure 5.**
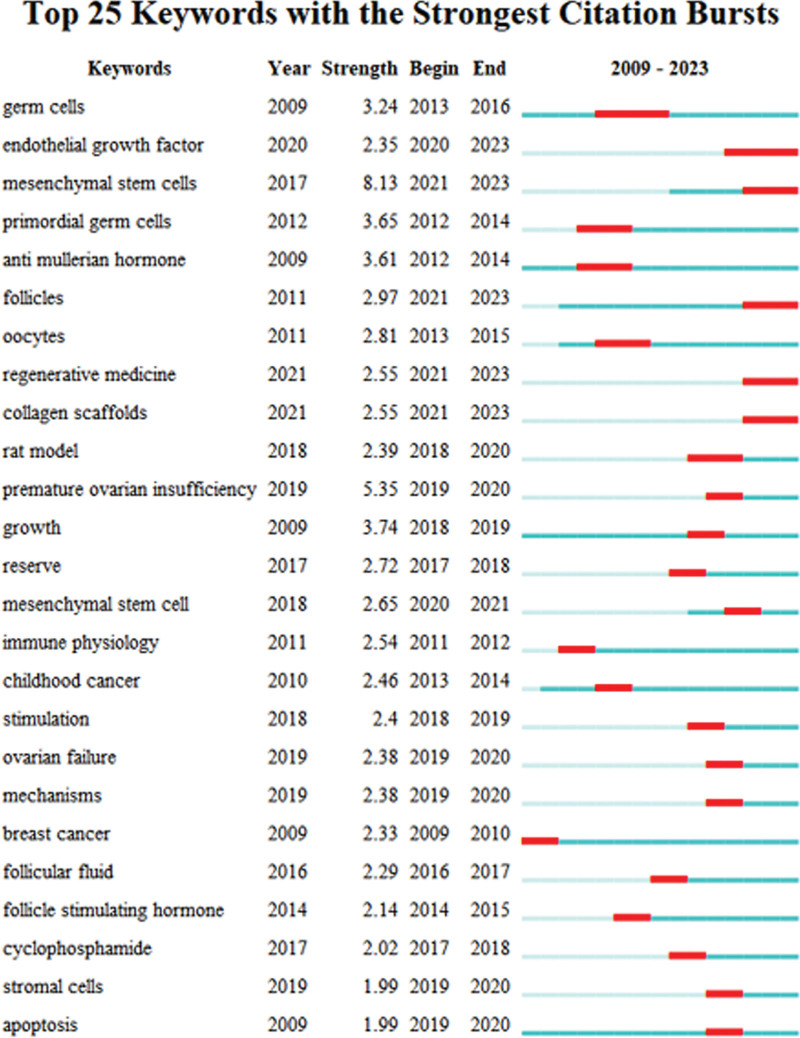
Keywords with the strongest citation bursts in stem cells research in POF. POF = premature ovarian failure.

A keyword importance during that period is shown by a keyword abrupt spike in usage frequency, known as “keyword emergence.” In POF research, the term “emergence” is used to explore the dynamic ideas and potential research questions that emerge in stem cells, to reflect ongoing or cutting-edge research nodes, and to help forecast research hotspots and trends in the future. According to Figure 5, strength denotes the degree of keyword emergence, Begin denotes the year the keyword first appeared within the scope of the study, End represents the year it stopped appearing, Blue block denotes the intensity unit annual time slice, and red block indicates the period corresponding to the keyword emergence. Between 2009 and 2023, early research hotspots were germ cells, primordial germ cells, anti-mullerian hormone, oocytes, immune physiology, childhood cancer, breast cancer, and folic stimulating hormone, followed by research hotspots such as rat model, POI, growth, reserve, stimulation, follicular fluid, ovarian failure, mechanisms, cyclophosphamide, stimulation, and other factors, More recently, research has shifted to endothelial growth factor, mesenchymal stem cells, regenerative medicine, collagen scaffolds, and extracorporeal cells, collagen scaffolds, extracellular vesicle, etc.

### 3.3. Analysis of authors

**Figure 6.**
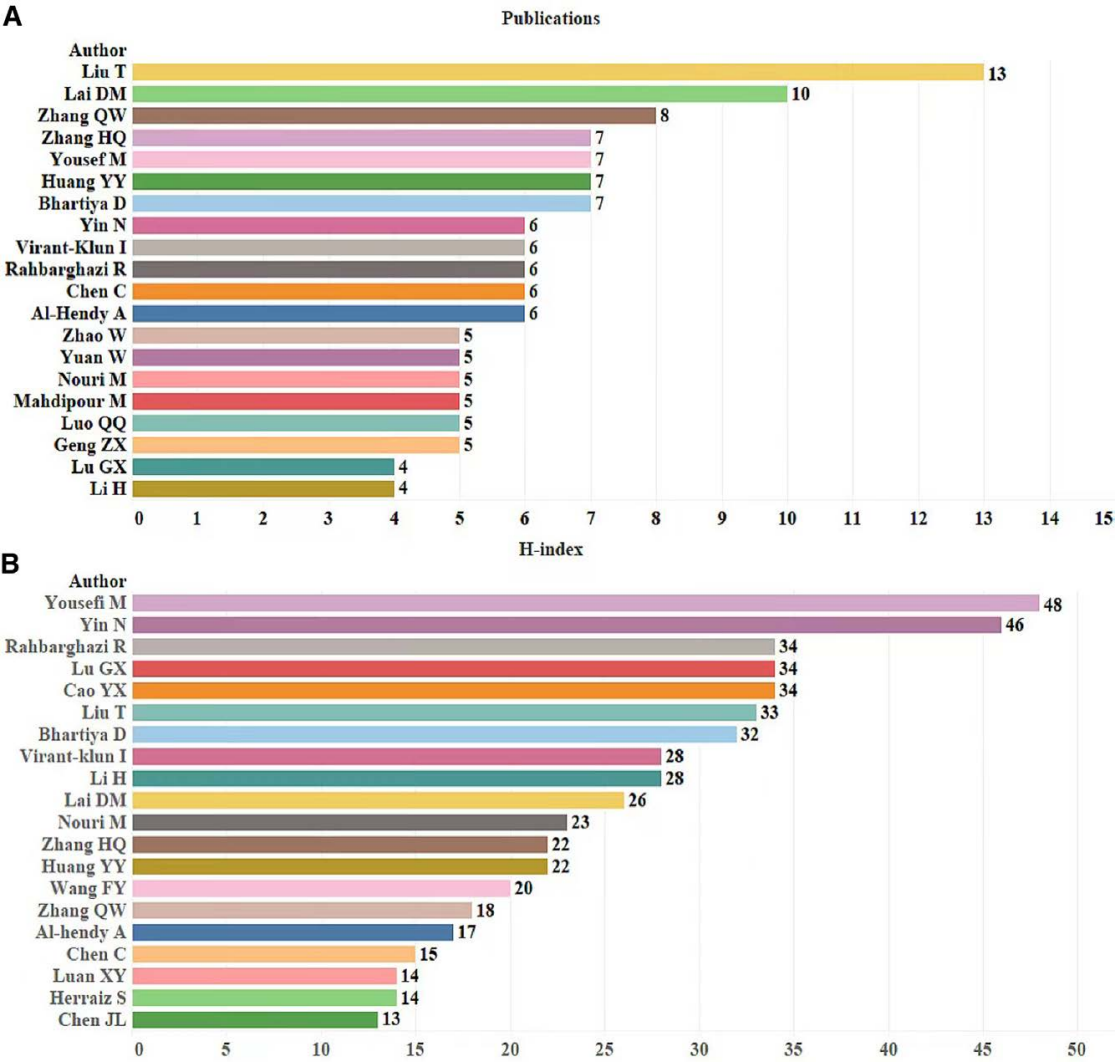
Author contributions to stem cells research in POF from 2009 to 2023. (A) Number of publications from different authors. (B) h-index of publications from different authors. POF = premature ovarian failure.

The author influence was evaluated in this study using the number of publications and the high index. The stem cells and POF study included 2038 authors. Liu T. has the most publications (n = 13), followed by Lai DM. (n = 12), Zhang QW. (n = 8), Yousefi M. (n = 7), and Huang YY. (n = 7) (Fig. 6A). Yousefi M. (H-index = 48), Yin N. (H-index = 46), Rahbarghazi R. (H-index = 34), Lu GX. (H-index = 34), Cao YX. (H-index = 34) were the top 5 writers according to the high index (Fig. 6B).

### 3.4. Analysis of intercountry/regional and interinstitutional cooperation

**Figure 7.**
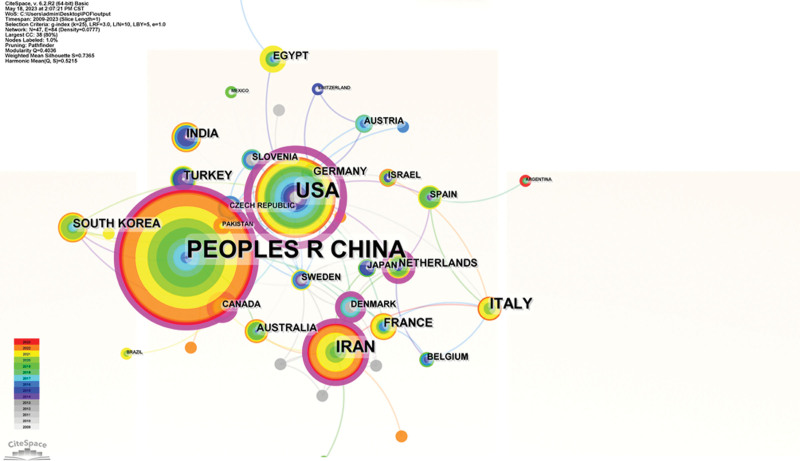
Analysis of countries involved in stem cells research in POF. POF = premature ovarian failure.

**Figure 8.**
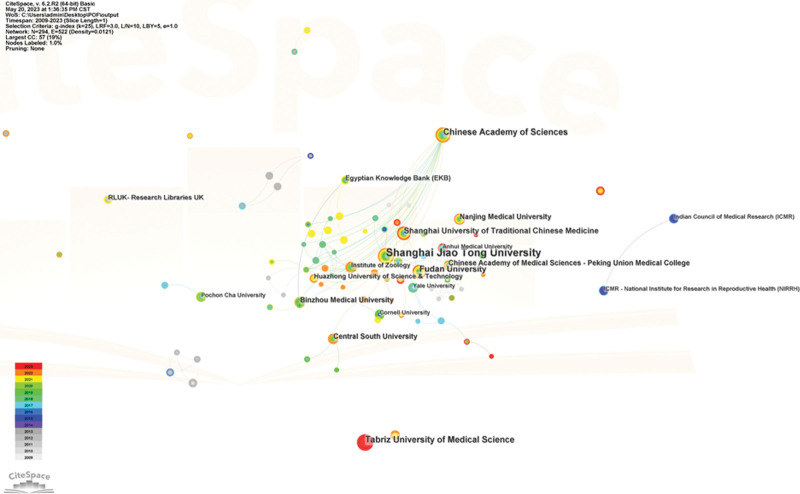
Analysis of institutions involved in stem cells research in POF. POF = premature ovarian failure.

The posting nations and institutions were visually analyzed and plotted using CiteSpace software. The size of each node in the graph represents the amount of literature produced on POF and stem cell research in that nation or institution, the connecting line between nodes indicates cooperation between 2 nations or institutions, and nodes with purple peripheries have a higher centrality. Each node in the graph represents a nation or institution. The US node has the largest purple circle among the studied nations (Fig. 7), has the highest centrality (centrality = 0.81), and is the leader in stem cell and POF research with a significant academic impact. Iran and China, which rank second and third globally behind the United States, also displayed large purple rings around the nodes with centralities of 0.15 and 0.13, respectively. According to statistics, China (171 articles) and the United States (74 articles), the leading countries studied, published 67.5% of the total papers. Among the research institutions, Shanghai Jiao Tong University (26 articles) had the highest number of publications, followed by Tabriz Univ Med Sci (14 articles) and Chinese Acad Sci (14 articles). Among the centers, Shanghai Jiao Tong Univ (centrality = 0.03) and Chinese Acad Sci (centrality = 0.03) were in the core position, followed by Fudan Univ (centrality = 0.02), see Figure 8.

### 3.5. Analysis of journals and co-cited academic journals

**Figure 9.**
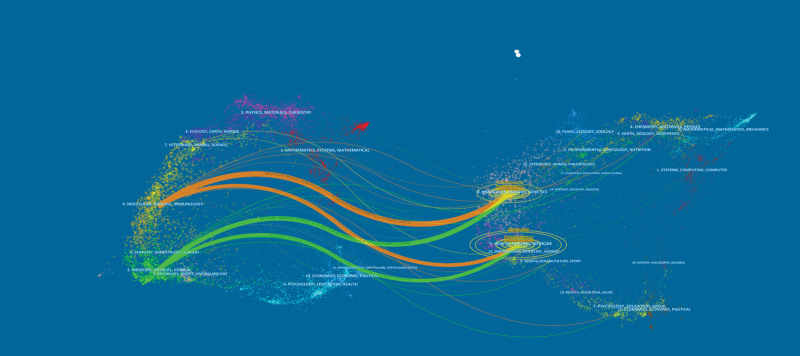
The dual-map overlay of journals involved in stem cells research in POF. The citing journals were located on the left of the map, while the cited journals were located on the right. Different colored lines represent different citation paths, and the width, z-value, and f-value size of the connecting paths are proportional to the number of citations. POF = premature ovarian failure.

All of the publications in this study were published in 193 different journals, according to Table 1; *STEM CELL RESEARCH THERAPY* had the most articles and influence (n = 30), followed by *JOURNAL OF OVARIAN RESEARCH* (n = 13), and *HUMAN REPRODUCTION* (n = 9). The dual-map overlay of journals (Fig. 9) shows the thematic distribution of journals. The citing journals are on the left side of the plot, and the cited journals are on the right side. The labels represent the disciplines covered by the journals. The colored lines depict the citation paths. There are 4 different citation paths. The 2 orange citation paths show that studies from Molecular/Biology/Immunology journals are frequently cited in studies from Molecular/Biology/Genetics and Health/Nursing/Medicine journals. The green path indicates that studies from Medicine/Medical/Clinical journals are frequently cited in studies from Molecular/Biology/Genetics and Health/Nursing/Medicine journals.

**Table 1:** The top 10 productive journals that published articles (N = 193).

Rank	Journal title	Country	Output [(%)]	IF(2022)	JCR(2022)
1	Stem Cell Research Therapy	England	30 [15.54]	8.079	Q1
2	Journal of Ovarian Research	England	13 [6.73]	5.506	Q1
3	Human Reproduction	England	9 [4.66]	6.353	Q1
4	Reproductive Sciences	United States	7 [3.63]	2.924	Q3
5	Stem Cell Reviews and Reports	United States	7 [3.63]	2.924	Q3
6	Biology of Reproduction	United States	6 [3.11]	4.161	Q2
7	Biomed Research International	United States	6 [3.11]	3.246	Q3
8	Frontiers in Cell and Developmental Biology	Switzerland	6 [3.11]	6.081	Q1
9	International Journal of Molecular Sciences	United States	6 [3.11]	6.208	Q1
10	Reproductive Biology and Endocrinology	England	6 [1.35]	4.982	Q1

### 3.6. Analysis of co-cited and high-centrality references

Table 2 summarizes the top 10 most cited references. One was entitled “Hippo signaling disruption and Akt stimulation of ovarian follicles for infertility treatment” (n = 154). Table 3 summarizes the top 10 references in terms of centrality. One article titled “Human amniotic fluid stem cells have a potential to recover ovarian function in mice with chemotherapy-induced sterility” had the highest centrality (Centrality = 0.21). By combining the number of citations and intermediary centrality, the 2 more influential and representative articles were “Human endometrial mesenchymal stem cells restore ovarian function through improving the renewal of germline stem cells in a mouse model of premature ovarian failure,” “Overexpression of miR-21 in stem cells improves ovarian structure and function in rats with chemotherapy-induced ovarian damage by targeting PDCD4 and PTEN to inhibit granulosa cell apoptosis,” which are not only cited more frequently but also intersect with other literature and play an essential role as a bridge in the network, indicating that these literature have crucial theoretical value and knowledge base, which are worth reading and studying.

**Table 2:** The top 10 cited references.

Rank	References	Title	Source	Yr	Country	Citations
1	Kawamura et al (2013)^[19]^	Hippo signaling disruption and Akt stimulation of ovarian follicles for infertility treatment	Proc Natl Acad Sci U S A	2013	Japan	154
2	Mclaughlin et al (2009)^[20]^	Awakening the oocyte: controlling primordial follicle development	Reproduction	2009	Australia	149
3	Lai et al (2015)^[21]^	Human endometrial mesenchymal stem cells restore ovarian function through improving the renewal of germline stem cells in a mouse model of premature ovarian failure	J Transl Med	2015	China	128
4	Xiao et al (2016)	Exosomal miR-10a derived from amniotic fluid stem cells preserves ovarian follicles after chemotherapy	Sci Rep	2016	China	117
5	Medeiros et al (2011)	Mir-290-295 deficiency in mice results in partially penetrant embryonic lethality and germ cell defects	Proc Natl Acad Sci U S A	2011	United States	117
6	Sun et al (2013)	Adipose-derived stem cells improved mouse ovary function after chemotherapy-induced ovary failure	Stem Cell Res Ther	2013	China	113
7	Liu et al (2014)	Transplantation of Human Menstrual Blood Stem Cells to Treat Premature Ovarian Failure in Mouse Model	Stem Cells Dev	2014	China	110
8	Takehara et al (2013)^[22]^	The restorative effects of adipose-derived mesenchymal stem cells on damaged ovarian function	Lab Invest	2013	Japan	102
9	Song et al (2016)^[23]^	Human Umbilical Cord Mesenchymal Stem Cells Therapy in Cyclophosphamide-Induced Premature Ovarian Failure Rat Model	Biomed Res Int	2016	China	97
10	Fu et al (2017)	Overexpression of miR-21 in stem cells improves ovarian structure and function in rats with chemotherapy-induced ovarian damage by targeting PDCD4 and PTEN to inhibit granulosa cell apoptosis	Stem Cell Res Ther	2017	China	96

**Table 3:** The top 10 high-centrality references.

Rank	References	Title	Source	Yr	Centrality
1	Lai et al (2013)^[24]^	Human amniotic fluid stem cells have a potential to recover ovarian function in mice with chemotherapy-induced sterility	BMC Dev Biol	2013	0.21
2	Kalich-Philosoph et al (2013)	Cyclophosphamide triggers follicle activation and “burnout”; AS101 prevents follicle loss and preserves fertility	Sci Transl Med	2013	0.21
3	Lai et al (2015)^[21]^	Human endometrial mesenchymal stem cells restore ovarian function through improving the renewal of germline stem cells in a mouse model of premature ovarian failure	J Transl Med	2015	0.17
4	Fu et al (2017)	Overexpression of miR-21 in stem cells improves ovarian structure and function in rats with chemotherapy-induced ovarian damage by targeting PDCD4 and PTEN to inhibit granulosa cell apoptosis	Stem Cell Res Ther	2017	0.16
5	Wang et al (2013)	Human amniotic epithelial cells can differentiate into granulosa cells and restore folliculogenesis in a mouse model of chemotherapy-induced premature ovarian failure	Stem Cell Res Ther	2013	0.16
6	Lai et al (2014)	Skin-derived mesenchymal stem cells help restore function to ovaries in a premature ovarian failure mouse model	PLoS One	2014	0.15
7	Abd-Allah et al (2013)	Mechanistic action of mesenchymal stem cell injection in the treatment of chemically induced ovarian failure in rabbits	Cytotherapy	2013	0.12
8	Elfayomy et al (2016)	Human umbilical cord blood-mesenchymal stem cells transplantation renovates the ovarian surface epithelium in a rat model of premature ovarian failure: Possible direct and indirect effects	Tissue Cell	2016	0.10
9	Yang et al(2020)^[25]^	Bone marrow mesenchymal stem cell-derived exosomal miR-144-5p improves rat ovarian function after chemotherapy-induced ovarian failure by targeting PTEN	Lab Invest	2020	0.08
10	Xiao et al (2014)	Amniotic fluid stem cells prevent follicle atresia and rescue fertility of mice with premature ovarian failure induced by chemotherapy	PLoS One	2014	0.08

## 4. Discussion

POF, a medically complex condition, arises from various underlying factors, including idiopathic, genetic, autoimmune, medical, and environmental causes, and is a challenging medical condition. In most situations, the method and cause of POF development are unknown.^[26]^ The percentage of idiopathic cases ranges from 74% to 90%. However, 4% to 30% of POF cases are believed to be caused by autoimmune disorders.^[5]^ Autoimmune diseases frequently accompany the progression of POF, and the most closely related one is thyroid dysfunction (Grave disease, hypothyroidism, and Hashimoto thyroiditis),^[27]^ followed by adrenal diseases, rheumatoid arthritis, Crohn disease, myasthenia gravis systemic lupus erythematosus, and multiple sclerosis. The reason for this phenomenon is that ovary cells are common targets of autoimmune system attacks.^[28]^ Endocrine and metabolic abnormalities are high-risk factors for POF. Based on the available literature, individuals treated for malignant tumors during childhood and adolescence have a higher risk of developing pharmacologic POF. Specifically, the risk is higher than that observed among those experiencing anxiety and depression (exceeding 27%) and among young diabetic women (approximately 2.5%)^[29,30]^; finally, environmental degradation serves as a significant and widespread potential risk factor for POF caused by smoking.^[31]^ These factors offer ample opportunities for stem cells, which possess immunomodulatory, anti-inflammatory, tissue repair, and organ reconstruction capabilities. Various tissue-derived stem cells can effectively restore ovarian function and follicle development in mouse models of chemotherapy-induced POF. Stem cells originate from diverse sources, including umbilical cord, umbilical cord blood, bone marrow, placenta, dental tissue, adipose tissue, menstrual blood, amniotic membrane, amniotic fluid, and others. They have emerged as the most promising candidates for regenerative medicine, highly regarded for their potential to repair tissue damage, suppress inflammatory responses, and enhance organ function.^[32]^ Their unique migratory, homing properties, regenerative function, tissue reconstruction, immunomodulatory, and paracrine effects help the body to be anti-fibrotic, anti-apoptotic, or pro-ovarian angiogenic. They are considered a prospective approach to treating POF caused by inflammatory and autoimmune diseases. In addition, the current breakthroughs in preclinical research and clinical trials of stem cells have brought new hope for treating POF.^[25,33]^ At present, the agreed upon mechanisms of POF repair by stem cells include restoration of damaged ovarian tissue structure, cell survival and secretion, and promotion of follicular growth through paracrine effects; immunomodulation to restore ovarian hormone levels, improve ovarian function and the immune microenvironment around the ovary, and improve inflammation; prevention of granulosa cell apoptosis, anti-fibrosis, and reduce ovarian damage. To the best of our knowledge, this paper represents the first attempt to present the knowledge map of international scientific publications related to stem cell and POF research from 2009 to 2023 through scientometric analysis, reviewing Fifteen years of research history, highlighting the present research status, hot spots, and frontiers, and focusing on future trends.

### 4.1. General information on stem cells research in POF

According to the annual publication and citation statistics (shown in Fig. 2), stem cell and POF research continues to gain interest and attention from researchers. In the initial phase from 2009 to 2011, Kaisa Selesniemi et al earlier proposed the ovarian surface epithelial stem cells in culture-like oocytes in vitro fertilization potential use.^[34]^ Stem cells effectively attenuate reproductive failure associated with POF and improve offspring survival, clarify that autoimmune diseases may play an essential role in the development of POF, and that stem cells help the ovaries secrete estrogen with breathtaking results, confirming that stem cell immunomodulation provides a new direction for POF prevention and treatment.^[35]^ Most early investigations mimicked the pathophysiology of human POF using in vitro or transgenic animals. Induced pluripotent stem cells (iPSCs), a novel strategy created by Shinya Yamanaka in 2006, helped to resolve the moral conundrum of simulating stem cell disorders and the issue of immune rejection. Parallel to this, the significant contributions of iPSCs have been evident in crucial in vivo studies and in vitro disease modeling, toxicity studies, medication development, and various other biomedical advancements. This also underscores the significance of the second phase, which exhibits a yearly increment in the number of publications and citations commencing in 2012. The third phase, from 2018 to 2023, focuses on subjects including germ cell destiny and alterations in gene expression profiles in stem cell transplanted models of POF.^[36,37]^ Numerous high-quality research on stem cells and POF have been published in the past 10 years due to the variety of stem cell sources, innovations in cell culture methods, iPSCs, and 3D bioprinting that have sped up the area development. The research conducted worldwide on this subject matter is constantly evolving, with a marked improvement in the quality of studies that has garnered widespread attention. This progress is reflected in the consistent production of approximately 60 publications per annum over the past 5 years, along with a steady rise in citation frequency. Regarding publication output, the top 3 nations were China, the United States, and Iran, as depicted in Figure 7. Asia, North America, Europe, and Oceania emerged as the most prolific continents among the top 10 nations, exhibiting substantial momentum in stem cell and POF research. Individual publication data is presented in Figure 6A, with Liu T. (n = 13) leading the way, followed by Lai DM. (n = 12), Zhang QW. (n = 8), Yousefi M. (n = 7), and Huang YY. (n = 7), who have contributed significant and pertinent material to advancing the field of research.

Additionally, the US has a significant advantage in this field, produces high-caliber academic articles, and occupies a prominent position in it, which may be related to the country large GDP and many financial allocations; the core author base has taken shape, and the global collaboration among publishing institutions and authors shows a crossover trend. Eight of the top 10 institutions in terms of number of publications were from China, with Shanghai Jiao Tong Univ having the most, and the rest from Iran (Tabriz Univ Med Sci) and England (RLUK- Research Libraries UK). In terms of centrality, the United States (Centrality = 0.81), Iran (Centrality = 0.15), and China (Centrality = 0.13) were in the core of research countries (Fig. 7); China ranked first globally in terms of the number of publications, with the research institution Shanghai Jiao Tong Univ (Centrality = 0.03) and Chinese Acad Sci (Centrality = 0.03)in the core, followed by Fudan Univ (Centrality = 0.02) (Fig. 8), leading institutions to publish high-level publications to deliver Chinese solutions globally. In addition, this study focuses mainly on the English publications of WOSCC and PubMed, which to some extent affects the accuracy of the analysis of centrality and core position for non-English speaking countries. In this study, Table 1 demonstrates that the journal *STEM CELL RESEARCH THERAPY* (n = 30) had the highest number of articles and impact, followed by *JOURNAL OF OVARIAN RESEARCH* (n = 13) and *HUMAN REPRODUCTION* (n = 9). This finding suggests that these journals are particularly interested in the research literature on stem cell therapy for POF and are helpful for researchers in choosing the best journals when publishing in this field. According to CiteSpace analysis, molecular and cell biology and immunology were the frequently used areas (Fig. 9). However, journals from different disciplines, such as genetics, nursing, and biochemistry, are also published in high volumes, indicating that stem cell and POF research is not limited to a single discipline and can be printed in journals with a broad audience. In addition, this field of molecular biology, immunology, nursing, and genetic genetics intersect closely. Exploring immune mechanisms, risk factor interventions, and psychological care may be core elements of POF prevention, treatment, and rehabilitation strategies.

### 4.2. Hot spots, frontiers and future of stem cell and POF research

This study demonstrates that emergent word analysis offers profound and invaluable insights into the evolving knowledge structure pertaining to POF and stem cell research. As evident from Figure 5, the top 25 frequently cited emergent terms within the realm of stem cell research on POF reveal an initial concentration on ultrastructural (electron microscopy) examination of germ cells in Nobox gene-deficient mice, primordial germ cells, oocytes, and the exploration of POF occurrence, progression, and associated factors (anti-Miller hormone, immunohistology, childhood cancer, breast cancer, FSH). Subsequently, the focus shifted toward establishing animal models and analyzing the structural relationships and operations of individual elements, particularly the interplay between stromal cells, apoptosis, and POF. In recent years, many studies have shown that stem cells can repair damaged tissues and improve inflammation to some extent, regulate apoptosis in their microenvironment, promote the development of stem cell fusion with POF using cell growth factors and collagen scaffolds, and restore ovarian function through stem cell proliferation, differentiation, and regeneration, further promoting the research capabilities of regenerative medicine. In addition, paracrine secretion of major mediators-extracellular vesicles from stem cells extracted from culture systems effectively circumvents immunocompatibility issues as a new and meaningful pharmacological strategy.^[38]^ More research should be conducted in this area, including beneficial information on drug delivery methods, combination drug use, and personalized and generic applications. In addition, the rapid development of nanotechnology provides new materials and techniques for stem cell investigation, long-term tracing of stem cell migration, proliferation, differentiation, pharmacokinetics, pharmacogenetics, cell fate and editing, and regeneration, monitoring of stem cell secreted chemical and biological substances and stem cell-microenvironment interactions, and regulating stem cell efficacy to accelerate their clinical translation.

This study analyzed high-frequency keywords and keyword-based clustering in 363 papers (as shown in Figs. 3 and 4). The high-frequency keywords are expression, failure, transplantation, women, mesenchymal stem cells, bone marrow, in vitro, fertility preservation, and mouse model. Differentiation, granulosa cells, activation, apoptosis, etc, were related to the topic of stem cells. Early on, primordial germ cells were isolated for mitosis using a flow cytometer in vitro in adult rat ovaries and human ovarian cortex tissues. Both in vivo and in vitro, these cells multiplied and displayed biological activity. Induced POF murine models elucidate the factors and mechanisms influencing ovarian function, effectively addressing the limitations of in vitro studies. The relationship between serum estrogen, FSH, antimelanocyte hormone, and granulosa cells and fertility was analyzed by animal models. It was proved that stem cells from various sources effectively improved hormone secretion and ovarian folliculogenesis in POF models, reduced ovarian apoptosis, and the fluorescence in situ hybridization technique traced the survival of transplanted stem cells in ovarian tissues without significant abnormal proliferation.^[19]^ Among the high-frequency keywords, chemotherapy and cancer are closely related to POF risk factors. The keywords fertility preservation and fertility suggest that protecting the fertility of POF patients is the focus of global efforts. Infertility caused by chemotherapy drugs to POF should not be underestimated, so it is important to develop cancer treatment strategies to systematically assess patients’ fertility preservation and reduce the risk associated with leading to ovarian decline to address the negative global population growth. The study data demonstrated that Angpt1 and Zcchc11 are important in measuring ovarian function. High expression of Angpt1 increases ovarian blood supply and promotes follicle growth; Zcchc11 upregulates IGF-1 expression, accelerates granulosa cell proliferation, reduces apoptosis, and enables rapid follicular cavity formation.^[20]^Of the 9 clustering modules (Fig. 4), #3 human endometrial mesenchymal stem cell, #7 noncoding RNA, and #8 cell fate regulator indicate various sources of stem cells and basal or regulatory-based noncoding RNA via pathways (e.g., #4 smad 9 signaling pathway) to study disease; #1 fertility preservation, #5 ovarian function, and #6 female sexual dysfunction are represented as thematic content features and categories indicating that stem cells open new possibilities for the POF.

As of April 7, 2021, Clinical Trials.gov reported that 133 clinical trials related to POF and stem cells had participants actively involved. Six of these studies were abandoned, 55 were finished, 10 were withdrawn, and another 10 were currently recruiting participants. About the use of stem cells as a treatment for POF, 17 research involving teenagers were recorded. Of these 17 trials, 4 were completed, 1 was abandoned, and 10 stem cell therapy trials for azoospermia were registered. Even though stem cell therapy gives the medical community hope and has excellent potential to treat POF, there are still unavoidable ethical issues with hESC and iPSC, safety concerns, non-essential differentiation, teratogenicity, and the potential for stem cells to spread tumors; practical study findings are affected by subject differences, the production process and quality control of stem cells, the choice of an appropriate transplantation time and dose, and other variables, which severely restrict the practical application of regenerative medicine. Therefore, a thorough assessment of crucial elements such as cell source and type, transport method, safety and efficacy, cell response to the implantation environment, and mechanism of action is urgently required. #0 POI is primarily characterized by early-onset POF, and genetic mutations significantly predict high POI risk. To address this, cryopreserved oocytes or stem cell repair for assisted reproduction offer a suitable ovarian environment for effective function. Understanding POI genetic and molecular underpinnings is crucial for comprehensive ovarian physiology knowledge and informed fertility guidance.

Most highly influential and central works were published earlier, presumably due to the delayed release of recent, high-quality literature that exhibited a relatively low citation rate and impact. Table 2 presents the 10 most frequently cited articles. The leading article reveals that suppressing Hippo signaling enhances the expression of downstream growth factors and stimulates follicle growth in a model of infertility caused by ovarian disease. Furthermore, the in vitro activation of Akt signaling by ovarian fragments holds promise in treating premature ovarian infertility.^[19]^ It is beneficial for middle-aged infertile women, sterilized cancer patients, and other scenarios where ovarian reserve is compromised, thereby facilitating the provision of more mature oocytes for embryonic development.^[20]^ In recent years, experimental animal models of stem cells have effectively restored damaged ovarian function, suggesting that stem cells provide suitable clinical strategies, new diagnostic biomarkers for regenerative medicine, and potential therapeutic targets for age-related human ovaries.^[21]^ The top 10 literature on intermediary centrality is presented in Table 3, with the majority of the early articles focusing on the purification or activation of eggs in vitro that have the same expression profile or function as primordial germ cells, challenging the long-held notion that the female mammalian ovary loses its capacity to produce oocytes before birth. However, scientific community members initially resisted this shift from conventional belief thinking. The purification of mitotically active cells with gene expression profiles similar to primordial germ cells that can be multiplied in vitro to create oocytes using a fluorescence-activated cell sorting-based approach for adult mouse ovary and human ovarian cortical tissue has been validated in the lab.^[39]^ Four of the top 10 central-ranking papers are about the transplantation of stem cells of different origins in animal models of POF. The transplanted mice gained weight, improved estrous cycles, and restored fertility while assessing ovarian histology, immunostaining, superovulation, and fertility in vitro fertilization, among other indicators. Stem cells remarkably convert the structure and function of damaged ovaries, offering new ideas and insights into the enormous clinical challenge of POF.^[23]^ The highly cited and centered literature shows a high interest in ovarian preservation. Different fertility preservation strategies such as pre-chemotherapy medication, ovarian translocation, embryo cryopreservation, oocyte vitrification, and ovarian tissue cryopreservation have been recently explored, and artificial ovaries, follicular stem cells, and drugs to prevent follicular loss have become promising approaches for the future. Transplantation of human umbilical cord-derived mesenchymal stem cells in an animal model restored hormonal secretion and folliculogenesis disorders in POF rats reduced ovarian apoptosis and traced transplanted stem cells. This protocol obtained not only live births after frozen ovarian tissue transplantation in adult patients but also provided reassuring evidence for the feasibility of prepubertal or pubertal age ovarian tissue implementation (first report of successful restoration of fertility after cryopreserved ovarian tissue transplantation in a 14-year-old purely syngeneic patient with sickle cell anemia before menarche).^[22,24,40]^ In addition, researchers have analyzed methods and criteria for fertility preservation from multiple perspectives and factors carried out earlier, sometimes reopening new debates on some of the already beneficial findings.^[41]^ Moreover, scholars have comprehensively assessed various methods and criteria pertaining to fertility preservation, considering various perspectives and factors, occasionally reigniting discussions surrounding previously advantageous discoveries.^[42]^

### 4.3. Limitation of this study

This study used a bibliometric approach to quantitatively analyze the existing stem cell and POF research literature. The dynamic development process, hotspots, research trends, and structural relationships between stem cells and POF provide a comprehensive guide for clinicians and scholars in this field. Inevitably, this study also has some limitations. Constantly updated databases may lead to discrepancies between search results, the number of publications included, and consideration of only those published in English articles. It may lead to some bias in the analysis, and from the study results, Chinese research institutions and scholars have done much work. However, the literature published in Chinese is not included in the analysis because there are only a few papers in the CNKI database, which cannot reach a certain number for visual analysis. Moreover, this study could not fully identify the role of the authors, who may be honorary or part-time. Despite these limitations, this study includes many papers on stem cell and POF research from 2009 to 2023 and can present the general status and trends in the field.

## 5. Conclusion

After conducting a visual analysis of the research hotspots, development status, and future trends in the field of Stem cells and POF using CiteSpace software, it has been established that stem cell immunomodulation, tissue repair, molecular mechanisms, and cell quality control of POF have experienced significant advancements in recent years. These advancements provide a solid foundation for further in-depth research and future topic selection in this crucial area.

## Author contributions

**Conceptualization:** Zhiguo Xu, Chao Liu.

**Data curation:** Chao Liu.

**Formal analysis:** Chao Liu.

**Funding acquisition:** Zhiguo Xu.

**Investigation:** Zhiguo Xu, Yi Zhu, Lefeng Liu, Chao Liu.

**Methodology:** Zhiguo Xu, Yi Zhu, Lefeng Liu, Chao Liu.

**Software:** Yi Zhu, Lefeng Liu, Chao Liu.

**Supervision:** Yi Zhu, Chao Liu, Zhilong Dong.

**Validation:** Yi Zhu, Lefeng Liu.

**Visualization:** Yi Zhu, Lefeng Liu.

**Writing – original draft:** Zhiguo Xu.

**Writing – review & editing:** Zhiguo Xu, Zhilong Dong.
